# Mechano-metabolic coupling in the tumor microenvironment: from mechanotransduction to protein lactylation and metastatic cascades

**DOI:** 10.3389/fimmu.2026.1991810

**Published:** 2026-10-27

**Authors:** Wenqing Zhang, Huimei Chen, Jingying Fan, Yingchun He, Jie Liu

**Affiliations:** 1 Hunan University of Chinese Medicine, Changsha, China; 2 The First Affiliated Hospital of Hunan University of Chinese Medicine, Changsha, China; 3 Hunan Provincial Engineering and Technological Research Center for Prevention and Treatment of Ophthalmology and Otolaryngology Diseases with Chinese Medicine and Protecting Visual Function, Hunan University of Chinese Medicine, Changsha, China; 4 Hunan Provincial Key Lab for the Prevention and Treatment of Ophthalmology and Otolaryngology Diseases with Traditional Chinese Medicine, Hunan University of Chinese Medicine, Changsha, China

**Keywords:** lactylation, mechanical signals, mechanotransduction, metabolic reprogramming, tumor metastasis, tumor microenvironment, YAP/TAZ

## Abstract

Aberrant mechanical signals within the tumor microenvironment (TME)—including increased extracellular matrix stiffness, elevated solid stress, heightened interstitial fluid pressure, and fluid shear stress—have been established as key drivers of malignant tumor progression and metastasis. These physical cues are sensed by mechanoreceptors on the cell membrane (Piezo1, TRP channels, and integrin-FAK complexes), transmitted intracellularly through Rho GTPases and cytoskeletal dynamics, and ultimately reprogram transcriptional programs via nuclear translocation of transcriptional co-activators such as YAP/TAZ. Mechanical signals also drive metabolic reprogramming in tumor cells, particularly enhancing aerobic glycolysis (the Warburg effect), which results in massive lactate accumulation. Landmark discoveries in recent years have revealed that lactate serves as a substrate to drive histone and non-histone lactylation, directly coupling metabolic status with epigenetic regulation. Histone lactylation (e.g., H3K18la, H3K9la) activates pro-metastatic transcriptional programs by remodeling chromatin accessibility, promoting epithelial–mesenchymal transition (EMT), immune evasion, and therapeutic resistance; non-histone lactylation directly modifies cytoskeletal proteins (CFL1, vimentin, TPX2) and transcriptional regulators (Snail, YY1, PRMT1), enhancing the metastatic execution capacity of tumor cells at multiple levels, including cellular mechanical properties, migratory ability, and signal transduction. Importantly, mechanical signals, metabolic reprogramming, and lactylation constitute a mechano-metabolism-lactylation positive feedback loop, forming a self-reinforcing vicious cycle that continuously fuels tumor metastasis. This review systematically outlines the types and mechanotransduction mechanisms of TME mechanical signals, mechanically driven metabolic and transcriptional reprogramming, the multifaceted functions of lactylation in tumor metastasis, and the ultimate execution of the metastatic cascade driven by this positive feedback loop, and discusses potential therapeutic strategies targeting mechanosensitive pathways, metabolic vulnerabilities, and lactylation-modifying enzymes.

## 1 Introduction

The tumor microenvironment (TME) has long been regarded as a passive soil upon which tumor cells depend, yet recent research has radically reshaped this view. The TME is not merely a reservoir of biochemical factors but rather a dynamic ecosystem replete with aberrant physical signals—including abnormally increased extracellular matrix (ECM) stiffness, accumulated solid stress, elevated interstitial fluid pressure (IFP), and fluid shear stress. These mechanical aberrations are now recognized as critical drivers of malignant tumor progression and metastasis (1, 2). Far from being simple byproducts of tumor growth, these physical cues constitute a complex signaling network that profoundly influences tumor cell fate, therapeutic responses, and metastatic potential (3). Over the past decade, mechanobiology has rapidly emerged at the forefront of oncology, revealing how physical forces are sensed, transduced, and integrated into intracellular biochemical programs, thereby reshaping gene expression, metabolic patterns, and cytoskeletal dynamics.

At the cell surface, mechanoreceptors such as Piezo1, transient receptor potential (TRP) channels, and integrin–FAK complexes serve as the first line of sentinels, converting mechanical inputs into chemical signals (4, 5). These sensors cooperate with cytoskeletal remodeling and Rho GTPase signaling to initiate a cascade of events that ultimately lead to the nuclear translocation of transcriptional co-activators, most notably YAP/TAZ, thereby orchestrating widespread transcriptional reprogramming (6). This reprogramming not only drives classical oncogenic pathways but also intimately intersects with cellular metabolism, giving rise to mechanically induced metabolic reprogramming, including the Warburg effect, remodeling of lipid and amino acid metabolism, and mitochondrial adaptation (7). Crucially, this mechanically driven Warburg effect results in profound lactate accumulation within the TME, creating a logical bridge between physical stress and metabolic output (8).

Linking this mechanically driven lactate pool to tumor progression represents a conceptual breakthrough in metastasis research, driven by the recent discovery of protein lactylation. Lactate, long considered a metabolic waste product of glycolysis, has now been demonstrated to serve as a substrate that directly drives lactylation of histones and non-histone proteins—a novel post-translational modification. This connection redefines how we understand physical forces in cancer. Mechanical cues do not just alter transient signaling cascades, they generate metabolic substrates that directly and stably rewire the epigenome and proteome. Histone lactylation (e.g., H3K18la) directly couples metabolic status with epigenetic regulation, positioning lactate as a signaling molecule that modulates chromatin accessibility and transcriptional programs (9–11). Non-histone lactylation targets cytoskeletal proteins, metabolic enzymes, and transcriptional regulators, modulating protein activity, stability, and subcellular localization to regulate cellular mechanical properties, migration, and invasion at multiple levels. Notably, a positive feedback loop exists between mechanical signals and lactate production. Mechanical stress drives glycolysis and lactate accumulation, while lactylation in turn potentiates mechanoresponsive pathways and cytoskeletal remodeling, continuously propelling tumor progression and metastasis (12, 13). The ultimate execution of this loop is manifested at each critical step of the metastatic cascade, including EMT, invadopodia-mediated ECM degradation, and the ability of tumor cells to deform and traverse physical barriers (14, 15). These steps are increasingly understood as processes co-regulated by mechanical and metabolic cues, with lactylation acting as a molecular rheostat that bridges extrinsic forces and intrinsic cellular adaptation.

Building upon these findings, we summarize recent progress and propose a hypothesis that a mechano-metabolic-lactyl positive feedback loop serves as the core engine driving the metastatic cascade, illustrating how TME mechanical signals are perceived by mechanoreceptors, transmitted through the cytoskeleton, and ultimately delivered to the nucleus to reprogram metabolism. We further discuss how this network drives metastatic progression, and envision therapeutic prospects targeting mechanosensitive pathways, metabolic vulnerabilities, and lactylation-modifying enzymes, in the hope of offering new insights for anti-metastatic strategies.

## 2 Mechanotransduction in the TME

The aberrant physical characteristics of the TME represent one of the key hallmarks of malignant progression, constituting a complex and heterogeneous mechanical signaling network. Beyond the classical increase in ECM stiffness, mechanical inputs in the TME also include elevated solid stress, IFP, and fluid shear stress (16–19). These physical cues act in concert to profoundly influence tumor cell fate and therapeutic responses.

### 2.1 Aberrant mechanical signals and physical barriers in the TME

#### 2.1.1 ECM stiffness and collagen crosslinking

Increased ECM stiffness is one of the most prominent physical features of solid tumors. This process primarily arises from hyperactivation of cancer-associated fibroblasts (CAFs) and excessive deposition and crosslinking of ECM proteins, particularly collagens (20–24). The underlying mechanisms include CAF-secreted extracellular vesicles rich in lysyl oxidase (LOX), which catalyzes collagen crosslinking, thereby directly enhancing ECM stiffness and stability (23, 25, 26). In multiple tumor types, such as hepatocellular carcinoma (HCC), colorectal cancer (CRC), and endometrial cancer (EC), tumor tissue stiffness is significantly higher than that of adjacent normal tissues and closely correlates with TNM stage, invasive/metastatic potential, and poor patient prognosis (27–30). For instance, in CRC, tumor stiffness measured by magnetic resonance elastography (MRE) positively correlates with collagen content and tumor progression (27). This increase in physical stiffness is not a static feature but a dynamic remodeling process, wherein CAFs continuously sense and respond to ECM mechanical changes via mechanoreceptors such as integrins, forming a positive feedback loop that promotes tumor progression (31).

#### 2.1.2 Solid stress, fluid pressure, shear stress, and their physical synergy in the TME

Rapid tumor proliferation and aberrant vascular networks jointly give rise to two other key mechanical abnormalities: elevated solid stress and IFP (32). Solid stress originates from physical compression of the tumor by surrounding normal tissues during rapid growth, as well as internal tension generated by ECM expansion (33). This compressive force can directly compress intratumoral blood and lymphatic vessels, leading to vascular collapse, hypoperfusion, and tissue hypoxia (20, 34). Meanwhile, the hyperpermeability of tumor vasculature and the lack of functional lymphatic drainage result in abnormal fluid accumulation in the tumor interstitium, generating elevated IFP (35–39). IFP is typically uniformly elevated throughout the tumor region and serves as a major physical barrier to the penetration of drugs, nanoparticles, and immune cells into the tumor core (36, 38, 40). High IFP not only hinders the delivery of therapeutic antibodies (e.g., trastuzumab) and nanomedicines but also directly promotes malignant behavior and immunotherapy resistance by affecting blood flow and immune cell infiltration (41, 42). For example, administration of a vascular normalizing agonist (dCNP) can effectively lower tumor IFP and improve drug delivery (43).

Dynamic mechanical inputs, particularly fluid shear stress, also play important roles in the TME (44–46). During circulation, circulating tumor cells (CTCs) must withstand shear stress generated by blood flow. Studies have found that tumor-resident microbiota can help CTCs resist fluid shear stress by remodeling the cytoskeleton (e.g., actin), thereby promoting distant metastatic colonization (44). Furthermore, the lymphatic system, as one of the major routes for tumor metastasis, imposes shear stress on tumor cells via lymph flow, a process involving activation of mechanosensitive ion channels such as Piezo1 (45). In liver cancer stem cells, fluid shear stress induces cell migration via RhoA/YAP mediate autophagy pathway (47). Notably, fluid shear stress does not act in isolation but is spatiotemporally coupled with ECM stiffness, solid stress, and IFP, collectively shaping the mechanical landscape of the TME (16). Dense ECM (high stiffness) impedes fluid reflux, further exacerbating IFP elevation, while elevated solid stress compresses blood vessels, exacerbating hypoxia, which in turn promotes CAF activation and ECM remodeling, forming a vicious cycle (24, 48, 49) (The heterogeneity and cooperativity of these distinct mechanical signals form a vicious cycle of malignant progression, as illustrated in **Figure 1**). Understanding the heterogeneity and complex cooperativity of these distinct mechanical signals is fundamental to elucidating TME mechanobiology and provides the necessary context for subsequent discussions on cellular mechanosensing and mechanotransduction (50).

**Figure 1:**
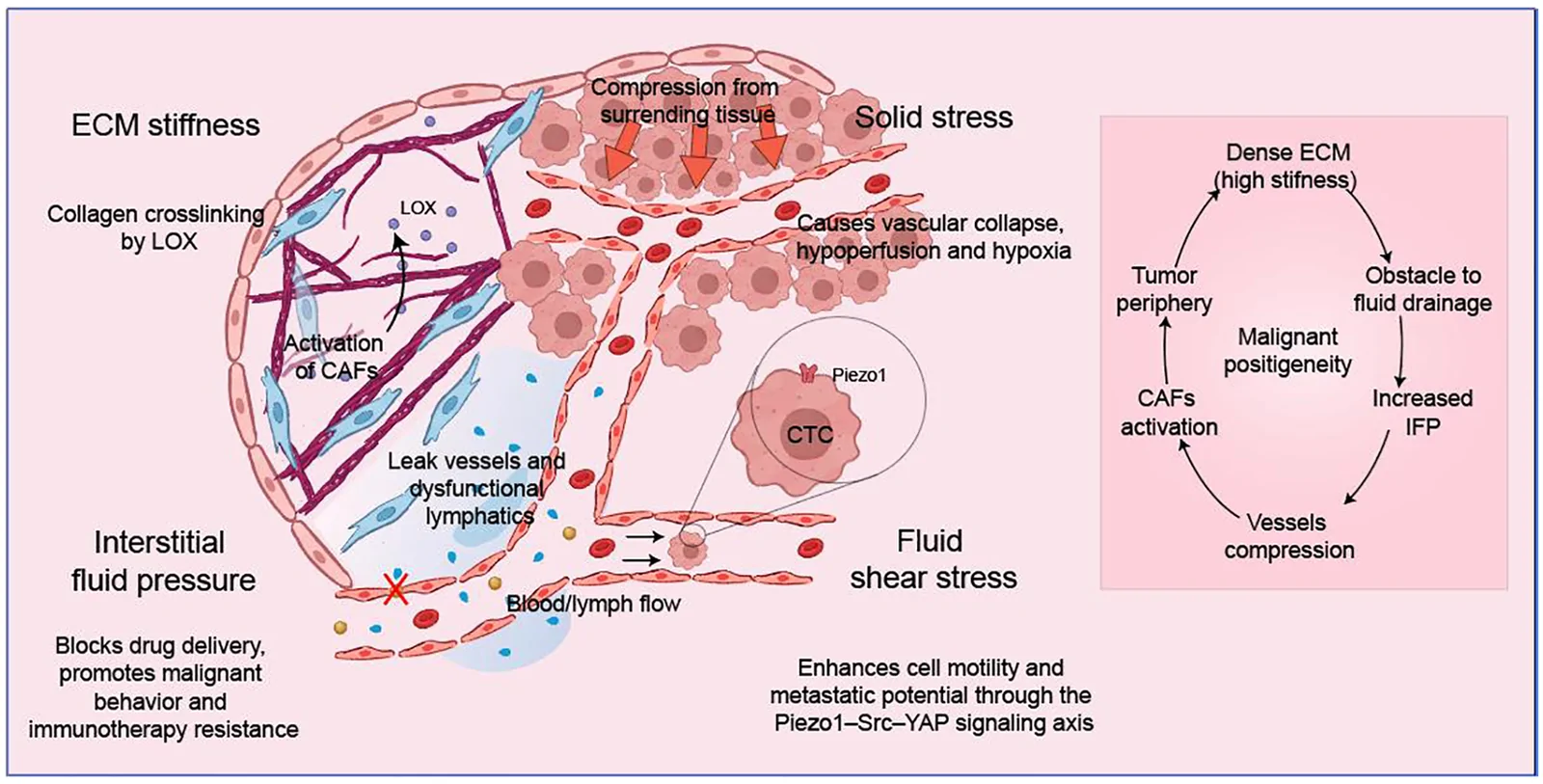
The multidimensional mechanical landscape of the tumor microenvironment and its malignant feedback loop. The tumor microenvironment (TME) is characterized by highly heterogeneous mechanical abnormalities. Left panel: Four primary mechanical inputs synergistically drive tumor progression (1). Extracellular matrix (ECM) stiffness: lysyl oxidase (LOX)-mediated collagen crosslinking and cancer-associated fibroblast (CAFs) activation lead to matrix rigidification (2). Solid stress: Rapid tumor proliferation and compression from surrounding tissue induce vascular collapse, hypoperfusion, and hypoxia (3). Interstitial fluid pressure (IFP): Leaky vessels and dysfunctional lymphatics elevate IFP, which blocks drug delivery and promotes malignant behavior as well as immunotherapy resistance (4). Fluid shear stress: Blood/lymph flow exerts shear stress on circulating tumor cells (CTCs), enhancing their motility and metastatic potential through the Piezo1/Src/YAP signaling axis. Right panel: A self-reinforcing vicious cycle of mechanical and biological cues. Dense ECM creates an obstacle to fluid drainage, escalating IFP and exacerbating vessel compression. This mechanically stressed and hypoxic environment further activates CAFs at the tumor periphery, driving continuous ECM stiffening and perpetuating malignant progression.

### 2.2 Core mechanoreceptors at the cell surface

Tumor cells as well as stromal and immune cells within the TME rely on various mechanoreceptors on the cell membrane to convert physical signals, such as ECM stiffness, shear stress, and tissue pressure, into intracellular biochemical signals. These core mechanoreceptors mainly include mechanosensitive ion channels (e.g., Piezo1, TRP family) and integrins along with their downstream focal adhesion kinase (FAK), and an emerging class of mechanoactivated G protein-coupled receptors (GPCRs), such as the angiotensin II type 1 receptor (AT1R), which together constitute the molecular basis for cells to sense and respond to the physical inputs of the microenvironment (51). Among them, Piezo1 is the most extensively studied mechanosensitive cation channel. Its activation triggers Ca2+ influx and initiates downstream signaling cascades. In pancreatic ductal adenocarcinoma (PDAC), pancreatic stellate cells (PSCs) sense stiffness gradients between tumor and normal tissues via Piezo1, guiding their directional migration toward stiffer regions (i.e., durotaxis). This process requires synergistic amplification by TRPC1 and TRPV4 channels, and the activity of all three must be maintained at moderate levels for optimal migration (52). In breast cancer cells, metastatic cells exhibit stronger integrin β1- and Piezo1-dependent collagen fiber remodeling and migration than non-metastatic cells, and knockdown of *Piezo1* disrupts directional mechanosensing (53). Moreover, under hypotonic stress, normal cells trigger actin cytoskeleton remodeling through Piezo1-mediated Ca2+ influx, which then activates the Na+/H+ exchanger NHE1 via ezrin, regulating cell volume and transcriptome. However, this mechanism is lost in tumor cells (e.g., HT1080, MDA-MB-231), suggesting that cancer cells adapt to the mechanical microenvironment by remodeling their volume regulation machinery (54).

In addition, Piezo1 functions in immune cells in a bidirectional and cell-type-specific manner. In CD4+ T cells, *Piezo1* deficiency promotes the production of pro-inflammatory cytokines such as IFNγ and IL-17, but reduces the effector memory T-cell pool, thereby failing to establish effective immunopathological responses *in vivo*, indicating that Piezo1 is essential for maintaining T-cell function and adaptive immunity (55). In tumor-infiltrating CD11b+ dendritic cells (DCs), conditional knockout of *Piezo1* instead enhances the cross-presentation ability of the cDC2A subset to antigens, promoting CD8+ T-cell antitumor responses, suggesting that mechanical regulation of DC function is subset-specific (56). Piezo1 is also involved in aging-related decline of mechanosensitivity in vascular smooth muscle cells (57), mediates swimming-induced endothelial Ca2+ signals in zebrafish larvae (58), regulates bone formation in chondrocytes (59), and exerts broad-spectrum antiviral effects through membrane fluidity remodeling (60). Collectively, these studies demonstrate that Piezo1 is a multifunctional mechanoreceptor whose functions are highly dependent on cell type and microenvironment.

Beyond Piezo1, TRP family channels such as TRPV4 and TRPC1 are also important mechanoreceptors. In the durotaxis of pancreatic stellate cells, after Piezo1 senses the stiffness gradient, its downstream signaling requires the involvement of TRPV4 and TRPC1 to fully amplify the Ca2+ signal. Blocking TRPC1/TRPV4 alone completely abolishes directional migration (52). Two-pore potassium channels (K2P) and calcium-activated potassium channels (KCa) indirectly regulate Ca2+ influx by maintaining the resting membrane potential and also play roles in tumor cell mechanotransduction (51). Notably, in addition to ion channels and integrins, growing evidence indicates that certain GPCRs themselves can also function as mechanoactivated sensors, thereby greatly expanding the repertoire of downstream biochemical pathways that can be triggered by mechanical force. AT1R is the most thoroughly studied example. Mechanical stretch can directly activate AT1R independently of angiotensin II, subsequently recruiting Gq/11 proteins and β-arrestin, and activating signaling cascades such as phospholipase C, ERK1/2, RhoA/ROCK, and EGFR transactivation, thereby converting physical stimuli into diverse intracellular signals (61). Other mechanosensitive GPCRs, such as GPR68, P2Y2, and various adhesion GPCRs, have also been shown to sense shear stress, matrix stiffness, and tissue tension, playing important regulatory roles in vascular and tumor microenvironments (62). Because GPCRs represent the largest family of druggable receptors, their mechanoactivation properties provide additional therapeutic entry points for intervening in TME mechanotransduction. Therefore, the mechanoreceptor system at the cell surface should be viewed as a multimodal synergistic network, in which ion channels, integrin–FAK complexes, and mechanosensitive GPCRs collectively convert physical signals into diverse intracellular biochemical responses (63). Integrins, as major mechanoadhesive molecules between cells and the ECM, cooperate with Piezo1 in regulating collagen fiber remodeling and migration in breast cancer studies (53), suggesting crosstalk between the integrin-focal adhesion axis and ion channel-mediated mechanical signaling pathways.

In summary, the mechanoreceptor system at the cell membrane is highly heterogeneous and synergistic. Piezo1 and TRP channels are responsible for rapidly sensing mechanical stimuli and generating Ca2+ signals, while integrin-FAK complexes mediate mechanical coupling and long-term adaptive responses between cells and the ECM. Mechanosensitive GPCRs such as AT1R and GPR68 further broaden the signaling landscape by activating G-protein- and β-arrestin-dependent cascades. The coupling of the cytoskeleton with ion transporters further integrates and amplifies the primary mechanical signals. The synergistic actions of these mechanoreceptors determine the response patterns of tumor cells and stromal cells to mechanical inputs from the TME, and constitute the upstream critical nodes for the activation of subsequent mechanotransduction pathways such as YAP/TAZ and Rho/ROCK.

### 2.3 Intracellular signaling hubs and cytoskeletal remodeling

After mechanical signals are captured by mechanoreceptors on the cell surface, they must be transmitted and integrated through intracellular signaling hubs, ultimately eliciting dynamic remodeling of the cytoskeleton to achieve adaptive responses to mechanical stimuli. This process involves the coordinated actions of Rho family GTPases, focal adhesion-associated proteins, the microtubule network, and various cytoskeletal regulators, and serves as the core driving force for tumor cell migration, invasion, and metastasis.

#### 2.3.1 Rho GTPases and actin cytoskeletal dynamics

Upon activation of mechanoreceptors at the cell membrane, mechanical signals are transmitted to and integrated by intracellular signaling hubs including Rho family GTPases, focal adhesion proteins, microtubule networks, and multiple cytoskeletal regulators, which ultimately trigger dynamic cytoskeletal remodeling and adaptive responses to mechanical stimulation (64). Within this network, Rho family GTPases such as RhoA, Rac1, and Cdc42 function as core molecular switches that cycle between inactive GDP-bound and active GTP-bound states, thereby converting mechanical inputs into spatiotemporally controlled actin polymerization, depolymerization, stress-fiber assembly, and cytoskeletal tension. For example, the mechanosensitive ion channel Piezo2 promotes cellular mechanobiological responses by regulating RhoA and the actin cytoskeleton (7). In confined microenvironments, the directional response of cells to fluid forces is also finely regulated by Rho-related signaling (65), high matrix stiffness accelerates the migration of HCC cells through the integrin β1/Plectin/F-actin axis, enhancing cytoskeletal tension and promoting cell motility (66). This Rho GTPase–actin regulatory axis is central to the mechanical adaptability of the cytoskeleton, which depends on the dynamics of actin polymerization and depolymerization. In models of cell migration within confined spaces, the dynamic changes in actin polymerization directly affect cell deformability and migration efficiency (67). Comparative studies of metastatic and non-metastatic breast cancer cells have shown that metastatic cells exhibit stronger cytoskeletal remodeling capacity, and this difference can serve as an important biomechanical indicator for assessing metastatic potential (68). The classic tumor suppressor p53 also inhibits tumor progression by regulating cytoskeletal remodeling, suggesting important crosstalk between tumor suppressor genes and mechanical signaling pathways (69).

#### 2.3.2 Roles of microtubules, lysosomes, and organelles in mechanotransduction

The microtubule network also participates in the transmission of mechanical signals. Recent studies have found that the mechanosensitive dynamics of lysosomes along microtubules regulate the emergence of leader cells during collective cell migration. Upon mechanical stimulation, the positioning and movement of lysosomes change, thereby affecting cell polarization and migratory behavior (70). Furthermore, studies using curved microfluidic confinement devices have shown that cell-shape-dependent nuclear mechanotransduction guides adaptive cell migration, closely correlating with nuclear envelope tension and YAP nuclear translocation (71). Cytoskeletal remodeling is an energy-consuming process, and there is tight coupling between mechanical signals and cellular metabolism. Studies on metastatic breast cancer cells in two-dimensional and three-dimensional microenvironments indicate that cytoskeletal reorganization patterns under different mechanical conditions are closely related to glycolytic and mitochondrial metabolic activities (72). In solid tumors, tumor cells often invade in collective migration modes. During collective migration, mechanical coupling between cells and dynamic regulation of junctional proteins are crucial. AQP3 increases intercellular adhesion in non-small cell lung cancer A549 cell spheroids by promoting exploratory cell protrusions (73). mechanochemical coupling and the distribution of contractile forces at cell-cell junctions during collective migration determine the morphology and directionality of the migrating front (74). Aligned fibrous scaffolds promote directional migration of breast cancer cells via caveolin-1/YAP-mediated mechanosensing (75). Multi-scale computational models provide theoretical frameworks for understanding cell and tissue dynamics (76).

In summary, cytoplasmic signaling hubs precisely regulate cytoskeletal dynamic remodeling through mechanisms involving Rho GTPases, actin polymerization/depolymerization, microtubule-dependent organelle transport, and mechano-metabolic coupling, driving both single-cell and collective cell migration and invasion. This is a critical link through which tumor cells adapt to and exploit the physical abnormalities of the TME to achieve metastatic dissemination.

### 2.4 Nuclear translocation of mechanical signals and transcriptional reprogramming

#### 2.4.1 Mechanoresponsive mechanisms of YAP/TAZ and regulation of nuclear translocation

Mechanical signals must ultimately be transmitted into the nucleus to reprogram gene expression by regulating the activity of transcription factors and co-activators. YAP/TAZ (Yes-associated protein/transcriptional coactivator with PDZ-binding motif), whose activity is primarily regulated by the Hippo pathway and its upstream mechanosensing components, is currently the most thoroughly studied mechanoresponsive transcriptional co-activator. Its nuclear translocation and activity regulation represent the core link connecting the extracellular mechanical environment to intranuclear transcriptional programs (77). On soft substrates or under low-tension conditions, the Hippo pathway kinases LATS1/2 phosphorylate YAP/TAZ, leading to cytoplasmic retention and degradation, whereas on rigid substrates or under high-tension conditions, YAP/TAZ are dephosphorylated and translocate into the nucleus, where they bind to TEAD family transcription factors and activate target gene expression (78–81). RAP2 is a key molecule that couples mechanical stimulation to the Hippo pathway, capable of responding to changes in cell membrane tension and regulating LATS1/2 activity (80). Piezo1-mediated Ca2+ signaling can promote matrix-stiffness-induced progression of clear cell renal cell carcinoma through the Calpain/YAP pathway (82). in bladder cancer, Piezo1/integrin β1 forms a positive feedback loop with Ca2+/YAP signaling to drive stiffness-dependent malignant progression (83). In addition, nuclear deformation directly affects YAP dynamics, in CAFs, nuclear deformation regulates YAP transcriptional activity by influencing its dynamic distribution (84). Adaptive changes in cell volume also control stem cell mechanotransduction, thereby affecting YAP/TAZ nuclear localization (85). Tension-activated YAP/TAZ can be transmitted via exosomes, forming intercellular signaling circuits (86).

#### 2.4.2 Functional diversity and pathway crosstalk of YAP/TAZ in cancer and stemness

In the TME, YAP/TAZ act as central mechanotransduction nodes that couple matrix stiffness to diverse malignant phenotypes and stemness programs. In HCC, heterogeneous matrix stiffness modulates the cancer stem cell-like phenotype via YAP/TAZ (87), niche stiffness sustains cancer stemness through phase separation of TAZ and NANOG (88). In lung adenocarcinoma, matrix stiffness coordinates cell proliferation and PD-L1 expression via YAP, directly linking mechanical signals to immune checkpoint regulation (89). In cutaneous squamous cell carcinoma, ECM stiffness drives malignant progression through YAP-dependent glycolysis (90). YAP/TAZ also play a central role in stem cell biology. Osteogenic versus adipogenic differentiation of mesenchymal stem cells (MSCs) is regulated by matrix stiffness, with YAP/TAZ playing a decisive role (91–93). Mechanical signals induce reprogramming of mature adipocytes through the YAP/TAZ-binding motif (94). In nucleus pulposus cells, YAP/TAZ cooperate with MRTF-A to respond to cell shape changes and maintain cellular phenotype (95). These studies reveal the conserved function of YAP/TAZ as universal mechanosensors in diverse cell fate decisions.

This functional diversity of YAP/TAZ is not achieved in isolation, it relies on extensive crosstalk with other signaling pathways, which allows YAP/TAZ to integrate mechanical cues with biochemical signals and thereby expand their regulatory repertoire in cancer and stemness. YAP/TAZ exhibit extensive crosstalk with signaling pathways such as TGF-β-Smad, Wnt/β-catenin, Notch, and ERα. In renal fibrosis, YAP/TAZ act as mechanical regulators of TGF-β-Smad signaling, synergistically promoting fibroblast activation and matrix deposition (96). Matrix stiffness promotes corneal fibrosis through the YAP/TAZ-β-catenin mechanotransduction pathway (97). In bone regeneration, YAP/TAZ and Notch signaling form a mechanoresponsive circuit that specifies specific endothelial cell subtypes (86). YAP/TAZ and ERα exhibit reciprocal regulation in mechanical and hormonal signaling in the skeletal system (98). Moreover, DDR1 forms a stiffness-responsive feedback autoregulatory loop through the YAP/TAZ axis (99), while the ROCK-TAZ signaling axis regulates mechanical tension-induced osteogenic differentiation of MSCs (91).

#### 2.4.3 Other nuclear mechanotransduction regulators and mechanisms

Beyond YAP/TAZ, other transcription factors and co-activators also directly respond to mechanical signals. RUNX2 is a key transcription factor in bone mechanobiology, regulating osteogenic differentiation (100). MRTF-A cooperates with YAP/TAZ in responding to cell shape changes (95). The MAPK pathway is also involved in matrix-stiffness-regulated osteogenic differentiation (101). In recent years, the nuclear envelope LINC complex (composed of SUN and KASH proteins) has been shown to directly connect the cytoskeleton to the nuclear skeleton, transmitting physical tension into the nucleus and affecting chromatin organization and gene expression (102). Nuclear compression can override soft matrix signals through actin-driven YAP mechanotransduction, regulating stem cell fate (103). In addition, contact inhibition, as an important mechanical feedback mechanism, is also regulated by mechanotransduction (104). Mechanical signals also affect gene expression through non-coding RNAs such as miRNAs (105). Engineered biomaterials and *in vitro* models have been widely used to study YAP/TAZ functions in cancer (50, 106). Targeting YAP/TAZ or their upstream regulators (e.g., LATS1/2) has emerged as a potential therapeutic strategy for antitumor and tissue regeneration applications (107, 108).

In summary, mechanical signals, through the YAP/TAZ-centric transcriptional co-activator network and auxiliary factors such as RUNX2 and MRTF-A, efficiently transmit information from the extracellular physical microenvironment to the nucleus, achieving transcriptional reprogramming with broad pathophysiological significance in tumor progression, stem cell differentiation, tissue fibrosis, and regenerative medicine (the mechanical transduction cascade from cell surface to nucleus is summarized in **Figure 2**).

**Figure 2:**
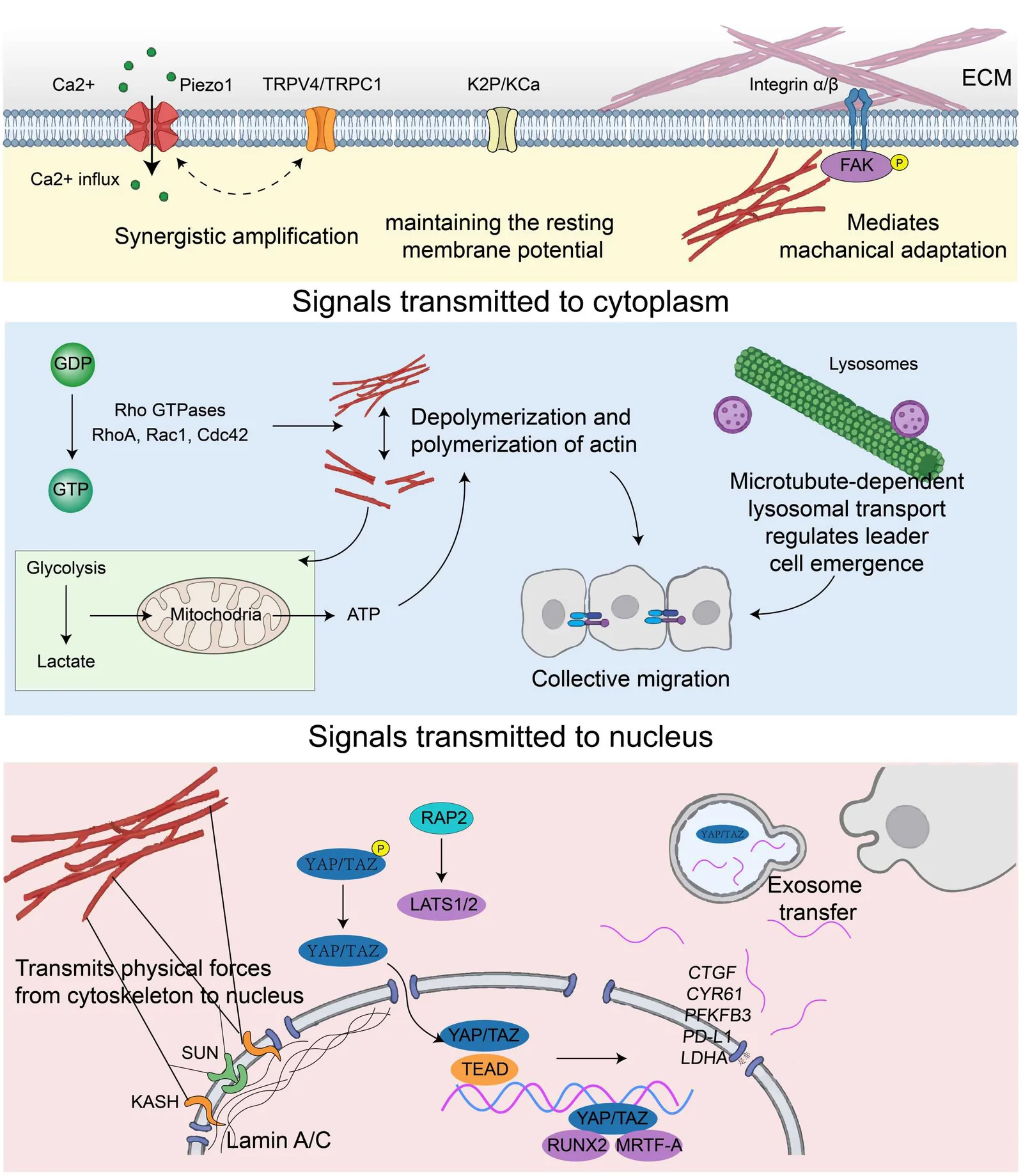
The outside-in mechanotransduction cascade: from cell surface mechanosensors to nuclear transcriptional reprogramming. The schematic illustrates the step-by-step transmission of mechanical signals across three cellular compartments. Top panel (Membrane): Piezo1 mediates Ca2+ influx, which is synergistically amplified by transient receptor potential vanilloid 4/transient receptor potential canonical 1 (TRPV4/TRPC1) channels, while two-pore potassium/calcium-activated potassium (K2P/KCa) channels help maintain the resting membrane potential. Concurrently, integrin-focal adhesion kinase (FAK) complexes mediate mechanical adaptation by interacting with the ECM. Middle panel (Cytoplasm): Signals are transmitted to the cytoplasm, where Rho guanosine-5’-triphosphatase (Rho GTPases; RhoA, Rac1, Cdc42), act as molecular switches to regulate the dynamic depolymerization and polymerization of the actin cytoskeleton. This cytoskeletal remodeling is tightly coupled with ATP production from mitochondria and glycolysis. Additionally, microtubule-dependent lysosomal transport regulates leader cell emergence, ultimately driving collective cell migration. Bottom panel (Nucleus): Mechanical signals are delivered into the nucleus to reprogram gene expression. Physical forces from the cytoskeleton are directly transmitted to the nucleus via the LINC complex, composed of Sad1 and UNC-84 (SUN) and Klarsicht, ANC-1, Syne homology (KASH) proteins and Lamin A/C. Meanwhile, the Ras-related protein 2/large tumor suppressor kinase 1/2 (RAP2/LATS1/2) axis regulates the phosphorylation state of YAP/TAZ. Dephosphorylated YAP/TAZ translocates into the nucleus, binding with TEA domain(TEAD), runt-related transcription factor 2(RUNX2), and myocardin-related transcription factor-A(MRTF-A) to activate the transcription of target genes. Tension-activated YAP/TAZ can also be transferred via exosomes to mediate intercellular signaling circuits.

## 3 Mechanics-driven metabolic reprogramming

Mechanical signals, via mechanoreceptors on the cell membrane and intracellular signaling hubs, ultimately act on nuclear transcription factors and co-activators to initiate or suppress the expression of metabolism-related genes, thereby achieving metabolic reprogramming in tumor cells. YAP/TAZ are central nodes connecting mechanosensing to metabolic transcriptional regulation, while upstream mechanoreceptors such as Piezo1 and FAK also regulate metabolic enzyme expression directly or indirectly (19, 109, 110).

### 3.1 Transcriptional regulation of metabolic reprogramming mediated by mechanical signals

#### 3.1.1 Transcriptional regulation of glycolytic genes by YAP/TAZ

Under rigid matrix or high mechanical tension, YAP/TAZ enter the nucleus and bind to TEAD transcription factors, directly upregulating the expression of multiple key glycolytic enzymes. Studies have shown that YAP/TAZ promote the transcription of phosphofructokinase-2/fructose-2, 6-bisphosphatase 3 (*PFKFB3*), a rate-limiting enzyme that controls glycolytic flux, its high expression is closely associated with matrix-stiffness-enhanced HCC growth and radioresistance (111, 112). In lung cancer, PFKFB3 regulates cancer stem cell properties through the Hippo pathway (113). Moreover, lactate dehydrogenase A (LDHA) is also a downstream target of YAP/TAZ, matrix stiffness upregulates LDHA via YAP/TAZ, thereby inducing lactylation and autophagy in PDAC cells (114). In mesothelioma, the YAP/TAZ/TEAD axis drives aerobic glycolysis, and natural compounds targeting this axis effectively inhibit tumor growth (115). Notably, YAP/TAZ transcriptional activity itself is subject to feedback regulation by glycolytic metabolites, forming a bidirectional mechanical-metabolic regulatory loop (116). In gastric cancer, the YAP/TAZ signaling pathway is also a key regulator of glycolysis (117, 118). Beyond this YAP/TAZ-centered transcriptional control, upstream mechanoreceptors and epigenetic modifiers indirectly remodel glycolytic gene expression. The Piezo1 mechanosensitive channel promotes chemoresistance in PDAC by mediating the expression of glycolysis-related genes in three-dimensional stiffness matrices (119). METTL3 regulates breast cancer glycolysis through m6A methylation of *LATS1* (120). USP7 supports pancreatic cancer glycolysis by stabilizing c-Myc (121). In addition, HDAC11 activity promotes MEK inhibitor escape in uveal melanoma (122), illustrating how epigenetic modifiers can shape therapy-resistant metabolic states. These mechanoreceptors and modifiers exhibit extensive crosstalk with YAP/TAZ signaling, collectively forming a regulatory network that amplifies glycolytic transcription under mechanical stress.

#### 3.1.2 Reprogramming of lipid, amino acid, and mitochondrial metabolism

Matrix stiffness and mechanical forces also reshape the lipid metabolic profile of tumor cells through transcriptional regulation. In HCC, stiff matrices induce the expression of stearoyl-CoA desaturase 1 (*SCD1*) via mechanosensitive pathways. SCD1 is a key enzyme in monounsaturated fatty acid synthesis, and its upregulation increases membrane fluidity, promoting HCC invasion and metastasis (29). In colorectal cancer, FUT2 reprograms fatty acid metabolism through YAP/TAZ signaling and SREBP-1, driving tumor metastasis (123). In pancreatic cancer, USP20-driven cholesterol metabolism synergizes with inflammatory signaling to promote stromal co-evolution (124). P-cadherin mechanically activates tumor-mesothelium metabolic coupling, promoting ovarian cancer metastasis (125). These findings indicate that YAP/TAZ are critical hubs integrating mechanical signals and transcriptional regulation of lipid metabolism.

The mechanical microenvironment also regulates amino acid utilization and mitochondrial oxidative phosphorylation (OXPHOS) through transcriptional mechanisms. In pancreatic cancer, the availability of amino acids (e.g., glutamine) is coordinated through YAP/TAZ-dependent transcriptional programs to sustain tumor growth and malignant phenotypes (126). In tumor-infiltrating regulatory T cells (Tregs), rigid matrices upregulate mitochondrial leucyl-tRNA synthetase 2 (*Lars2*) via YAP, enhancing OXPHOS and thereby maintaining the immunosuppressive function of Tregs (127). In addition, high-fat diet-induced ECM remodeling regulates metabolism-related gene expression through activation of Piezo1-dependent mitochondria-ER contacts (128). CLIC1 constructs a network linking matrix stiffness to the Warburg effect, promoting pancreatic cancer growth through transcriptional regulation (129). YEATS2, as a matrix-stiffness-induced epigenetic regulator, drives HCC progression through the TGFBR2/TAZ/AKT pathway (130). Moreover, Focal adhesion kinase (FAK) influences energy metabolism in glioblastoma stem cells by regulating the expression of genes involved in glycolysis and glutamine oxidation (131), further linking upstream mechanoreceptors to amino acid and mitochondrial metabolic programs. These results reveal the fine control of lipid, amino acid, and mitochondrial metabolism by mechanical signals via transcriptional regulation.

#### 3.1.3 Bidirectional mechano-metabolic feedback and concluding remarks

Mechanical regulation of metabolic gene transcription is not unidirectional, metabolic changes can also feed back to modulate the transcriptional output of mechanical signals. YAP/TAZ activity itself is regulated by nutrient-sensing pathways such as AMPK and mTOR (132). In *NF2*-deficient tumor cells, inhibition of YAP/TAZ induces metabolic and signaling rewiring, exposing targetable metabolic vulnerabilities (133). In confined cellular microenvironments, there is tight coupling between mechanotransduction and metabolic adaptation (134). Matrix stiffness orchestrates NET formation and mTOR-driven metabolic reprogramming in lung cancer (135). Hyperglycemia induces pancreatic cancer metabolic reprogramming and EMT through the YAP/TAZ-Hedgehog axis (136). These findings reveal the central role of bidirectional mechano-metabolic feedback in tumor progression.

In summary, mechanical signals, via transcriptional co-activators such as YAP/TAZ and downstream signals from upstream sensors like Piezo1 and FAK, precisely regulate the expression of genes in key metabolic pathways including glycolysis, lipid synthesis, amino acid metabolism, and mitochondrial respiration. This network not only drives malignant phenotypes but also provides a theoretical basis for anticancer therapies targeting mechanosensitive metabolic pathways (137–139).

### 3.2 Spatial and allosteric regulation of metabolic enzymes via cytoskeletal tension

The cytoskeleton not only serves as the structural foundation for cellular mechanics but also directly interacts with metabolic enzymes, enabling spatial organization and allosteric regulation of metabolic activity. Traditionally, glycolytic enzymes were thought to be uniformly distributed in the cytosol, but substantial evidence indicates that multiple glycolytic enzymes, including aldolase, PFK, and GAPDH, can specifically bind to F-actin and microtubules, forming metabolic microcompartmentation that spatially coordinates energy production with cytoskeletal functions (140–147).

#### 3.2.1 Physical binding of glycolytic enzymes to the cytoskeleton and its reversibility

Aldolase is the most thoroughly studied actin-binding metabolic enzyme. Biochemical and structural studies have shown that aldolase binds to specific interfaces on F-actin via its C-terminal region, with binding constants modulated by ionic strength, pH, and molecular crowding (148–151). Besides aldolase, PFK, GAPDH, and lactate dehydrogenase (LDH) can also interact with actin or microtubules, forming multienzyme complexes (144, 152). In foundational studies using yeast, plant systems, and non-transformed mammalian cells, In yeast, plants, and mammalian cells, this binding is highly dynamic. Carbon source withdrawal or osmotic changes can cause glycolytic enzymes to dissociate from the cytoskeleton, accompanied by actin reorganization (153, 154). This indicates that cytoskeleton-metabolic enzyme coupling is a rapid response mechanism for cells to adapt to environmental mechanical and nutritional changes. When contextualized within oncology, Hsp90 regulates the regional distribution of glycolytic enzymes (including aldolase and PFK) in the cytoplasm, promoting gastric cancer cell metastasis and stemness maintenance (155). TPM4 condenses glycolytic enzymes and facilitates actin reorganization (156). In addition, aldolase interacts with ARNO to regulate cell morphology and acidic vesicle distribution (157), and mediates the association of GLUT4 vesicles with F-actin (158).

#### 3.2.2 Mechanical regulation of enzyme-cytoskeleton dissociation and metabolic flux reprogramming

Mechanical tension and downstream signaling pathways can precisely control the binding state of glycolytic enzymes to the cytoskeleton, thereby modulating glycolytic flux. Classical studies in both non-tumor physiological models and malignant systems have revealed that the PI3K/AKT signaling pathway promotes the release of aldolase from the actin cytoskeleton through phosphorylation. Upon release, aldolase catalytic activity is significantly enhanced, promoting glycolysis and tumor cell survival (159). Similarly, LOXL2-mediated deacetylation of aldolase alters its binding affinity to actin, inducing metabolic reprogramming and tumor progression (160). During axonal retraction in neural physiological models, ATP produced by local glycolysis directly drives actomyosin contraction (161). Under mechanical strain, the co-chaperone BAG3 coordinates protein synthesis and autophagy through spatial regulation of mTORC1 (162). Under hyperosmotic stress, TPM4 condenses glycolytic enzymes and promotes actin reorganization (156). Local anesthetics and calmodulin antagonists can induce dissociation of PFK and aldolase from the cytoskeleton in melanoma cells, altering glycolytic rates (163, 164). In cardiomyocytes and skeletal muscle, glycolytic enzymes localize to costameres, providing energy for contraction (165).

Of particular note, mechanical signals not only regulate metabolic flux through enzyme–cytoskeleton dissociation but can also directly control the plasma membrane localization of metabolite transporters. In adherent epithelial cells, fluid shear stress can induce enrichment of glucose transporter 1 (GLUT1) at cell–cell junctions, thereby enhancing glucose uptake to meet the high energy demands of cytoskeletal remodeling (166). This process depends on the coordinated action of E-cadherin and actomyosin. Shear stress drives GLUT1 recruitment to E-cadherin adhesion complexes through non-muscle myosin IIA-mediated contractility (167). In medulloblastoma, cerebrospinal fluid shear stress is sensed through the mechanosensitive ion channel Piezo2, which then drives actomyosin contractility-dependent GLUT1 plasma membrane recruitment, enhancing glucose uptake and promoting metastasis (168). Although these physiological and somatic paradigms illuminate the biophysical principles of force-induced metabolic reprogramming, future studies must determine how these localized energetic shifts are rewired under the chronic mechanical stresses unique to malignant solid tumors. (The dynamic interaction between glycolytic enzymes and the cytoskeleton, which is critically modulated by mechanical tension, is depicted in **Figure 3**).

**Figure 3:**
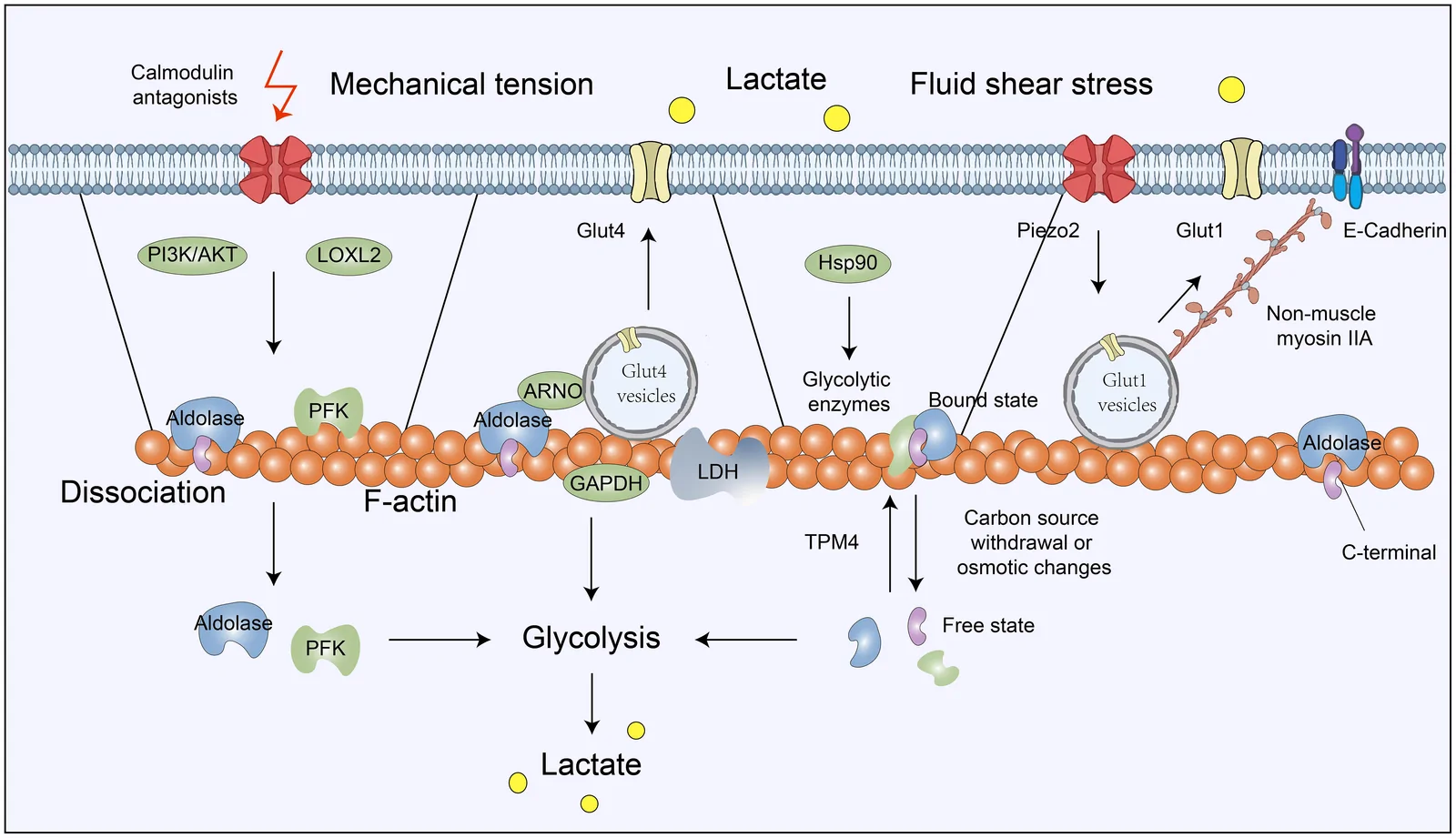
Spatial and allosteric regulation of glycolytic enzymes via F-actin cytoskeletal tension. Glycolytic enzymes, including aldolase, phosphofructokinase (PFK), GAPDH, and lactate dehydrogenase (LDH), can physically bind to F-actin to form metabolic microcompartments. Aldolase binds specifically to F-actin via its C-terminal region. This binding is highly dynamic: environmental cues such as carbon source withdrawal, osmotic changes, or the action of tropomyosin 4 (TPM4) can cause these enzymes to shift between a bound and a free state. Heat shock protein 90 (Hsp90) regulates the regional distribution of these glycolytic enzymes, while aldolase also interacts with ADP-ribosylation factor nucleotide-binding site opener (ARNO) to mediate the association of GLUT4 vesicles with F-actin. Importantly, mechanical tension and its downstream signaling cascades tightly fine-tune such protein interactions. Under fluid shear stress, the coordination between E-cadherin and the actomyosin complex promotes GLUT1 recruitment to cell-cell junctions and the plasma membrane, boosting glucose uptake to fuel energy consumption for cytoskeletal remodeling. Furthermore, the PI3K/AKT signaling pathway, lysyl oxidase-like 2 (LOXL2), and calmodulin antagonists can induce the dissociation of aldolase and PFK from the actin cytoskeleton. Upon release, the catalytic activity of these enzymes is significantly enhanced, leading to upregulated glycolysis and massive lactate production, thereby rapidly adjusting metabolic flux to meet the energy demands of migrating cells.

#### 3.2.3 Moonlighting functions of metabolic enzymes and mechanosensing

In addition to their catalytic functions, metabolic enzymes such as aldolase also directly participate in cytoskeletal dynamics and mechanotransduction as actin-binding proteins. Aldolase can interact with WASP family proteins and the Arp2/3 complex, regulating actin polymerization and cell motility (158, 169–171). While mechanistic insights into these non-canonical roles have frequently been pioneered in parasite models (171, 172), specialized immune subsets (173), or neural development (174). they provide compelling conceptual templates for cancer biology. In carcinoma cells, such moonlighting activities allow metabolic enzymes to act as dual-function mechanosensors. Changes in cytoskeletal tension can simultaneously affect metabolic flux and cytoskeletal dynamics by altering the localization and binding state of metabolic enzymes. In terms of spatial distribution, the elastic distribution of actin filaments determines cell migratory behavior (175), and perinuclear actin mechanical regulation of cell volume also affects metabolism (160). Vitamin D_3_ reduces glycolysis and invasiveness in breast cancer cells while increasing cell stiffness, suggesting that it may act by modulating enzyme-cytoskeleton interactions (176). This mechano-metabolic coupling mode is not limited to metabolic enzymes themselves but also extends to the level of metabolite transporters. GLUT1 recruitment to E-cadherin adhesion complexes under shear stress further demonstrates that mechanical signals can precisely deploy glucose uptake capacity to mechanically active regions by remodeling adhesion junctions and the actomyosin network, providing local energy support for cytoskeletal remodeling and mechanical work.

In summary, cytoskeletal tension directly regulates the spatial distribution and binding state of glycolytic enzymes, enabling rapid and localized reprogramming of metabolic activity to provide energy support for cell migration and mechanical work, though the exact translational boundaries from non-neoplastic model organisms to human TME pathophysiology remain an active area of investigation.

### 3.3 Mechanically-induced Warburg effect

Aberrant mechanical signals in the TME, including increased ECM stiffness, solid stress, and fluid shear stress, have been shown to directly drive metabolic reprogramming in tumor cells toward aerobic glycolysis (i.e., the Warburg effect). ECM stiffness activates the mTOR signaling pathway, coordinating neutrophil extracellular trap (NET) formation and metabolic remodeling to promote lung cancer progression (135). In pancreatic cancer, hypoxia and ECM stiffness induce the expression of ubiquitin-specific protease *USP7*, which stabilizes c-Myc protein through deubiquitination, thereby upregulating glycolysis-related genes, enhancing glucose uptake, lactate production, and extracellular acidification rate, thus supporting the Warburg effect and tumor growth (121). Furthermore, during the transition from pulmonary fibrosis to lung cancer, the metabolic heterogeneity of macrophages is also regulated by mechanical signals from the microenvironment (177). These findings indicate that mechanical forces not only act as physical barriers affecting drug delivery but also actively induce the Warburg effect by directly regulating key signaling molecules such as c-Myc and mTOR, providing energy and biosynthetic precursors for rapid proliferation and metastasis of tumor cells in a stressful microenvironment. Targeting mechanically sensitive glycolytic regulatory nodes (e.g., the USP7/c-Myc axis) may represent a novel strategy for improving solid tumor therapy.

## 4 Lactylation and the mechano-metabolic coupling mechanisms in tumor metastasis

### 4.1 The metamorphosis of lactate from metabolic waste to post-translational modification donor

For a long time, lactate was regarded as a terminal metabolite of glycolysis, accumulating abundantly in the TME. However, recent studies have overturned this traditional view, revealing that lactate is not only an energy substrate but also an important signaling molecule and a precursor for post-translational modifications (PTMs) (10, 178–182). In 2019, Zhang et al. first reported histone lysine lactylation (Kla) as a novel epigenetic modification, directly catalyzed by lactate-derived lactyl-CoA, thereby directly coupling cellular metabolic status with gene expression regulation (9, 183). This milestone discovery marked a fundamental metamorphosis of lactate’s role from a metabolic waste product to a key metabolite in epigenetic regulation.

#### 4.1.1 Lactate: from metabolic waste to signaling molecule

Driven by the Warburg effect, tumor cells preferentially undergo glycolysis even under aerobic conditions, producing large amounts of lactate. The traditional view considered lactate merely a metabolic byproduct, but the lactate shuttle hypothesis has revealed the intercellular and intracellular transport and utilization of lactate (181, 184, 185). Lactate accumulates in the TME via monocarboxylate transporters (MCTs), not only acidifying the microenvironment but also participating as a signaling molecule in angiogenesis, immune evasion, and metastasis (181, 182). In recent years, lactate has been found to serve as a substrate directly driving histone and non-histone lactylation, tightly linking metabolic reprogramming to epigenetic regulation (9, 179, 186).

#### 4.1.2 Discovery of lactylation and its enzymatic regulation

Histone lactylation was initially identified on core histones (H3, H4) in mouse and human cells, with H3K18la and H4K12la being the earliest discovered sites (9, 183). Lactylation depends on intracellular lactate levels and the generation of lactyl-CoA. Under hypoxic or high-glycolysis conditions, tumor cells produce abundant lactate, part of which is converted intracellularly to lactyl-CoA as a substrate for histone lactyltransferases. Multiple “writer” enzymes, such as p300 and KAT8, have been identified to catalyze lactylation (187–189), while “eraser” enzymes such as HDAC1-3 and SIRT3 possess delactylase activity (178, 190). The dynamic balance of lactylation is finely regulated by these enzymes and is also influenced by the availability of lactyl-CoA (178, 191, 192).

#### 4.1.3 Universality and functional diversity of lactylation

Through high-throughput lactylome analysis, researchers have identified thousands of lactylation sites covering histones and non-histone proteins in various tumors, including HCC, colorectal cancer, gastric cancer, pancreatic cancer, and oral squamous cell carcinoma (193–198). For example, in HCC, 9, 256 out of 9, 275 lactylation sites are located on non-histone proteins, preferentially enriched in metabolic pathways (199). In oral squamous cell carcinoma, lactylation under hypoxia is significantly enriched in glycolytic enzymes and spliceosomal proteins (197). These findings indicate that lactylation is extensively involved in metabolic adaptation, proliferation, metastasis, immune regulation, and therapy resistance (178, 200). In addition, non-histone lactylation (e.g., of moesin, eEF1A2, YTHDC1, ABCF1, DCBLD1, NSUN2, PYCR1, etc.) exerts multiple regulatory functions by altering protein stability, activity, or subcellular localization (201–205). Therefore, lactylation constitutes a new language connecting metabolic reprogramming to cellular functional regulation, representing a highly promising frontier in tumor biology.

### 4.2 Histone lactylation and pro-metastatic transcriptional remodeling

Histone lactylation, by directly regulating the transcription of metastasis-related genes, serves as the core epigenetic mechanism by which mechano-metabolic coupling drives tumor metastasis. Multiple histone lactylation sites (e.g., H3K18la, H3K9la, H3K14la, H3K27la, etc.) have been shown to activate the expression of pro-metastatic genes, promoting EMT, cell migration, invasion, and distant colonization (15, 206–226).

#### 4.2.1 Histone lactylation drives transcription of metastasis-related genes

In multiple solid tumors, histone lactylation promotes metastatic progression by activating specific target genes. In pancreatic cancer, H3K18 lactylation upregulates *CXCL1* expression, promoting neutrophil infiltration and immune evasion (214), H3K9 lactylation promotes colorectal cancer progression by upregulating the cholesterol transporter *GRAMD1A* (213). In HCC, histone lactylation enhances metastatic capacity by promoting NSF1-mediated ferroptosis resistance and Rab7A lactylation-driven exosome biogenesis (201, 221). *VHL* inactivation-triggered histone lactylation forms a positive feedback loop with PDGFRβ signaling, driving clear cell renal cell carcinoma progression (211). In gastric cancer, H3K18la-mediated *VCAM1* expression promotes metastasis via the AKT/mTOR/CXCL1 axis (219). YY1 lactylation activates *FBXO33* transcription, which in turn degrades p53 to promote gallbladder cancer metastasis (15). In lung cancer, H3K18 lactylation promotes proliferation and metastasis through the H3K18la/VCAM1 axis and the KCNK1/LDHA positive feedback loop (220, 226). Moreover, GPR37 enhances glycolysis and histone lactylation via the Hippo pathway, driving colorectal cancer liver metastasis (212). *KRAS* mutation promotes colorectal cancer metastasis by upregulating *GRAMD1A* via H3K9la (213). These studies reveal the universality of histone lactylation as a core driver of pro-metastatic transcriptional reprogramming.

#### 4.2.2 Histone lactylation regulates EMT and cytoskeletal remodeling

Histone lactylation plays a central role in EMT regulation. In diabetic kidney disease, H3K14la drives EMT through the KLF5 pathway (184). CAF-secreted LOX promotes PD-L1 expression via histone lactylation and regulates tumor EMT through TGFβ/IGF1 signaling in gastric cancer (218). Hyperglycemia induces pancreatic cancer metabolic reprogramming and EMT through the YAP/TAZ/Hedgehog axis (196). KCNK1 promotes breast cancer EMT by activating LDHA and upregulating H3K18la (210). Notably, there is bidirectional regulation between histone lactylation and cytoskeletal remodeling. Hypoxia-induced PRMT1 lactylation drives vimentin arginine symmetric dimethylation, promoting cytoskeletal reorganization and metastasis (207). These findings indicate that histone lactylation not only regulates transcription factor networks but also indirectly reshapes cellular mechanical properties by influencing the expression of cytoskeleton-related genes.

#### 4.2.3 Histone lactylation mediates immune evasion and therapy resistance

Histone lactylation plays a critical role in remodeling the immune status of the TME. In glioblastoma, lactate upregulates *CD47* via H3K18 lactylation, inhibiting macrophage phagocytosis (227). In pancreatic cancer, histone lactylation drives *SPP1*+ macrophage polarization, *CXCL1* expression, and NET formation, promoting an immunosuppressive microenvironment (214, 216). In HCC, lactylation-driven NUPR1 promotes immunosuppression of tumor-infiltrating macrophages (228). Histone lactylation regulates M2 macrophage polarization via SRSF10 (229). In lung cancer, H3K9 lactylation induces CD8+ T cell dysfunction (217). H3K18 lactylation promotes tumor immune evasion through B7-H3 expression (224). STAT5 upregulates PD-L1 by promoting histone lactylation, driving immunosuppression in acute myeloid leukemia (221). Furthermore, histone lactylation contributes to therapy resistance. In ovarian cancer, H3K9 lactylation activates *RAD51* and *BRCA2* expression, promoting homologous recombination repair and platinum resistance (229). Targeting histone lactylation (e.g., inhibiting p300 or LDHA) has emerged as a new strategy to enhance immunotherapy and overcome drug resistance (230–234).

In summary, histone lactylation drives a series of malignant phenotypes—including EMT, immune evasion, and distant colonization—by remodeling chromatin accessibility and transcriptional programs, serving as a critical bridge between mechanically induced Warburg effect and tumor metastasis (The epigenetic mechanism by which histone lactylation drives pro-metastatic gene transcription is illustrated in **Figure 4**). Targeting histone lactylation and its regulatory enzymes (p300, HDACs, KAT8, etc.) holds promise as a novel therapeutic strategy for inhibiting tumor metastasis.

**Figure 4:**
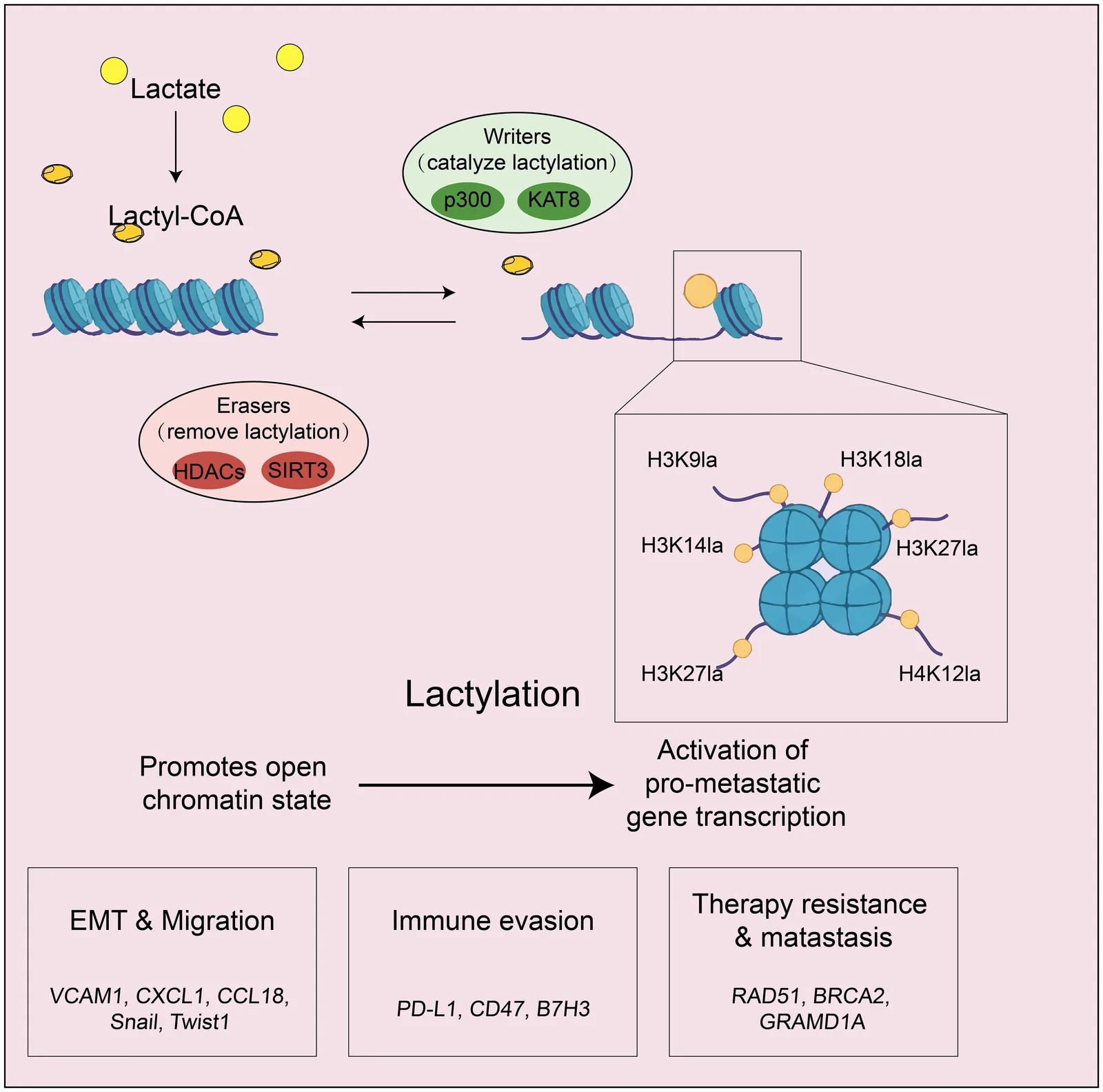
Histone lactylation drives pro-metastatic transcriptional remodeling. Intracellular lactate is converted to lactyl-coenzyme A (lactyl-CoA), serving as the direct substrate for histone lactylation(Kla; lysine residues modified by lactyl groups, denoted as site-la, e.g., H3K9la means lactylation at lysine-9 of histone H3). This dynamic epigenetic modification is orchestrated by “writers” [e.g., p300 and lysine acetyltransferase 8 (KAT8)] that catalyze the addition of lactyl groups, and “erasers” [e.g., histone deacetylases (HDACs) and sirtuin 3(SIRT3)] that remove them. The accumulation of specific histone lactylation marks promotes an open chromatin state, leading to the robust transcriptional activation of pro-metastatic genes. This epigenetic reprogramming empowers tumor cells with multifaceted malignant capabilities, facilitating EMT and migration, driving immune evasion, and conferring therapy resistance along with enhanced metastatic potential.

### 4.3 Non-histone lactylation: core mechanisms reshaping the cytoskeleton and metastatic execution

Non-histone lactylation represents another important dimension of lactylation in tumor biology. Unlike histone lactylation, which regulates gene transcription epigenetically, non-histone lactylation directly modifies metabolic enzymes, cytoskeletal proteins, signal transducers, and transcriptional regulators, rapidly and precisely modulating malignant phenotypes—particularly cytoskeletal dynamics and metastatic execution functions—by altering their stability, activity, subcellular localization, or interaction networks (199, 235–239). High-throughput lactylome analysis has revealed the universality of this modification across various tumors (199, 240, 241). Crucially, non-histone lactylation operates not as isolated biochemical events, but as a functional bridge in the mechano-metabolic-lactyl loop, converting mechanically driven lactate accumulation into functional, structural, and transcriptional adaptations.

#### 4.3.1 Lactylation of cytoskeletal and structural proteins directly executing mechanical remodeling

Direct modification of cytoskeletal and structural proteins by lactylation serves as the immediate downstream execution arm of the mechano-metabolic-lactyl loop, directly translating lactate signaling into altered cell stiffness and motility. In nasopharyngeal carcinoma, circCDYL enhances glycolysis and lactate accumulation by stabilizing *LDHA* mRNA, thereby inducing lactylation of the actin-binding protein CFL1 (cofilin-1) at lysine 22 (K22). CFL1 lactylation reduces cell adhesion and stiffness, promoting tumor cell proliferation, invasion, and migration (242). Beyond microfilaments, microtubule dynamics and intermediate filaments are similarly modulated. In HCC, TPX2 (targeting protein for Xklp2) undergoes lactylation at lysine 249 (K249), catalyzed by CBP and removed by HDAC1. TPX2 lactylation disrupts its binding to protein phosphatase 1 (PP1), enhances AURKA kinase activity, and promotes cell cycle progression and tumor growth (243). In pancreatic cancer, the microtubule-associated protein NUSAP1 is lactylated, forming a NUSAP1/LDHA/glycolysis/lactate positive feedback loop that continuously drives the Warburg effect and metastasis (244). In addition, in breast cancer, hypoxia-induced PRMT1 lactylation enhances its methyltransferase activity, catalyzing symmetric dimethylation of vimentin at arginine 64 (R64) to drive vimentin intermediate filament assembly (207). These structural modifications collectively provide the physical basis for cell deformation, matrix traversal, and mechanical adaptation during the metastatic cascade.

#### 4.3.2 Lactylation of transcriptional regulators driving mechano-responsive and EMT programs

Lactylation of transcriptional regulators serves as an integrative node that links metabolic outputs back to mechano-responsive gene expression, amplifying the feedback loop. In pancreatic cancer, RHOF promotes *PKM2* transcription via c-Myc to enhance glycolytic lactate production, which in turn induces lactylation of Snail. Lactylated Snail undergoes nuclear translocation to activate the EMT program, driving cell migration and invasive properties (12). In HCC, lactylation of ABCF1 at lysine 430 (K430) promotes its nuclear translocation, where it binds to the *KDM3A* promoter to upregulate its expression, activating the KDM3A/H3K9me2/HIF1 axis and forming a lactate/ABCF1 lactylation/HIF1/lactate positive feedback loop that drives tumor growth and lung metastasis (188). By modifying these master regulators, lactylation ensures that initial mechanical stress induces a prolonged transcriptional state favorable for invasion.

#### 4.3.3 Lactylation of metabolic enzymes and DNA repair factors supporting energy demand and genomic stability

In addition to structural and transcriptional reprogramming, non-histone lactylation targets metabolic enzymes and DNA repair factors, providing the energetic sustenance and genomic integrity required to endure mechanical stress during metastasis. High-throughput studies show that lactylation is highly enriched in metabolic enzymes, such as PFKP in oral squamous cell carcinoma under hypoxia (241), and in TCA cycle and glycolytic enzymes in HCC (199), establishing an autoregulatory metabolic network that sustains high glycolytic flux under physical strain. Concurrently, lactylation modulates DNA repair and survival mechanisms during passage through challenging physical microenvironments. In ovarian cancer, lactylation of RAD51 at lysine 73 (K73) enhances homologous recombination repair, conferring resistance to platinum therapy (245). In breast cancer, RCC2 lactylation (K124) facilitates its recruitment of SERBP1 to stabilize *MAD2L1* mRNA, promoting cell proliferation (246). Furthermore, delactylation of PD-L1 (K189) promotes its nuclear translocation, upregulates *SQLE* expression, enhances cholesterol synthesis, and accelerates liver cancer growth (247). Together, these modifications optimize metabolic flux, DNA repair, and immune evasion, protecting metastasizing tumor cells as they encounter physical barriers.

In summary, non-histone lactylation acts as a versatile executor of the mechano-metabolic-lactyl loop by systematically categorizing its substrates into structural proteins (CFL1, vimentin, TPX2, NUSAP1) and transcriptional regulators (Snail, ABCF1, etc.), metabolic enzymes (PFKP), and DNA repair factors (RAD51). Targeting non-histone lactylation (e.g., CFL1 K22la, TPX2 K249la, etc.) and their upstream/downstream signaling bridges holds great therapeutic promise for disrupting the positive feedback loop and halting tumor metastasis (248, 249).

### 4.4 The hypothesis of mechano-metabolic-lactyl feedback loop

There is complex bidirectional crosstalk among mechanical signals, cellular metabolic reprogramming, and post-translational modifications (especially lactylation), forming a self-reinforcing positive feedback loop that continuously drives malignant tumor progression. Mechanical forces induce metabolic reprogramming through cytoskeletal remodeling, while metabolic products (particularly lactate) in turn enhance cellular responses to mechanical signals by modifying cytoskeletal proteins and key components of mechanosignaling pathways, constituting the core mechanism of this loop (250, 251).

On one side of this proposed framework, direct experimental observations confirm that ECM stiffness and mechanical tension can directly trigger cytoskeletal reorganization, which in turn regulates glycolytic flux. Specifically, *in vitro* studies have shown that topology-mediated inhibition of cytoskeletal spreading induces metabolic reprogramming in bone marrow mesenchymal stem cells, upregulating paracrine-related factors (252). During development, dynamic remodeling of the actin network is functionally coupled to metabolic reprogramming, and cytoskeletal strain can modulate glycolytic flux and influence cell fate decisions through nuclear mechanotransduction pathways (253). Cyclic mechanical stretch regulates lens epithelial cell senescence in an intensity-dependent manner, involving the integrin-cytoskeleton-ECM axis together with p53 signaling and metabolic reprogramming (254). Moreover, increased matrix stiffness directly promotes F-actin polymerization and cell stiffening, while disruption of actin polymerization reverses mechanical damage (255). These direct observations collectively establish that mechanical inputs directly guide the reshaping of metabolic status through cytoskeletal dynamics.

On the reciprocal side, direct experimental evidence also shows that the products of metabolic reprogramming—particularly lactate—can feed back to regulate the cytoskeleton and mechanosignaling systems. High levels of lactate in the TME not only serve as an energy substrate but also directly alter the function of cytoskeletal proteins and mechanoreceptors through lactylation. For example, lactylation has been directly observed to modify cytoskeleton-associated proteins such as CFL1, vimentin, and TPX2, affecting cell stiffness and migratory capacity (see Section 4.3). Simultaneously, mechanical stress can induce the release of lysosomal proteases in a process dependent on an intact cytoskeleton, suggesting the existence of a mechano-lysosome-metabolism axis (256). Functional nanoplatforms mediate caspase cascades by remodeling cytoskeletal assembly, mechanosensitive channel activation, and metabolic reprogramming, thereby enhancing antitumor immunity (250), further illustrating the intrinsic coupling between mechanical cues and metabolic responses.

In summary, we propose a hypothesis wherein a positive feedback loop consisting of mechanical input, cytoskeletal remodeling, metabolic reprogramming (enhanced glycolysis, lactate accumulation), lactylation, reprogramming of the cytoskeleton and mechanosignaling pathways may operates continuously in tumors to amplify metastatic potential. This hypothesized loop is conceived to not only amplifies the biological effects of initial mechanical signals but also endows tumor cells with the ability to adapt to the dynamic mechanical microenvironment (integrated into the schematic framework presented in **Figure 5**). Crucially, it must be emphasized that while the individual legs of this cascade are supported by direct experimental observations—predominantly derived from isolated *in vitro* models—direct *in vivo* evidence validating the complete, self-sustaining closed loop during cancer metastasis remains limited. Thus, the holistic integration of this loop represents a rational indirect inference based on current discrete findings. Targeting key nodes in this proposed loop (e.g., lactate transport, lactylation enzymes, or cytoskeletal regulators) holds promise for breaking the positive feedback and providing new strategies for cancer therapy.

**Figure 5:**
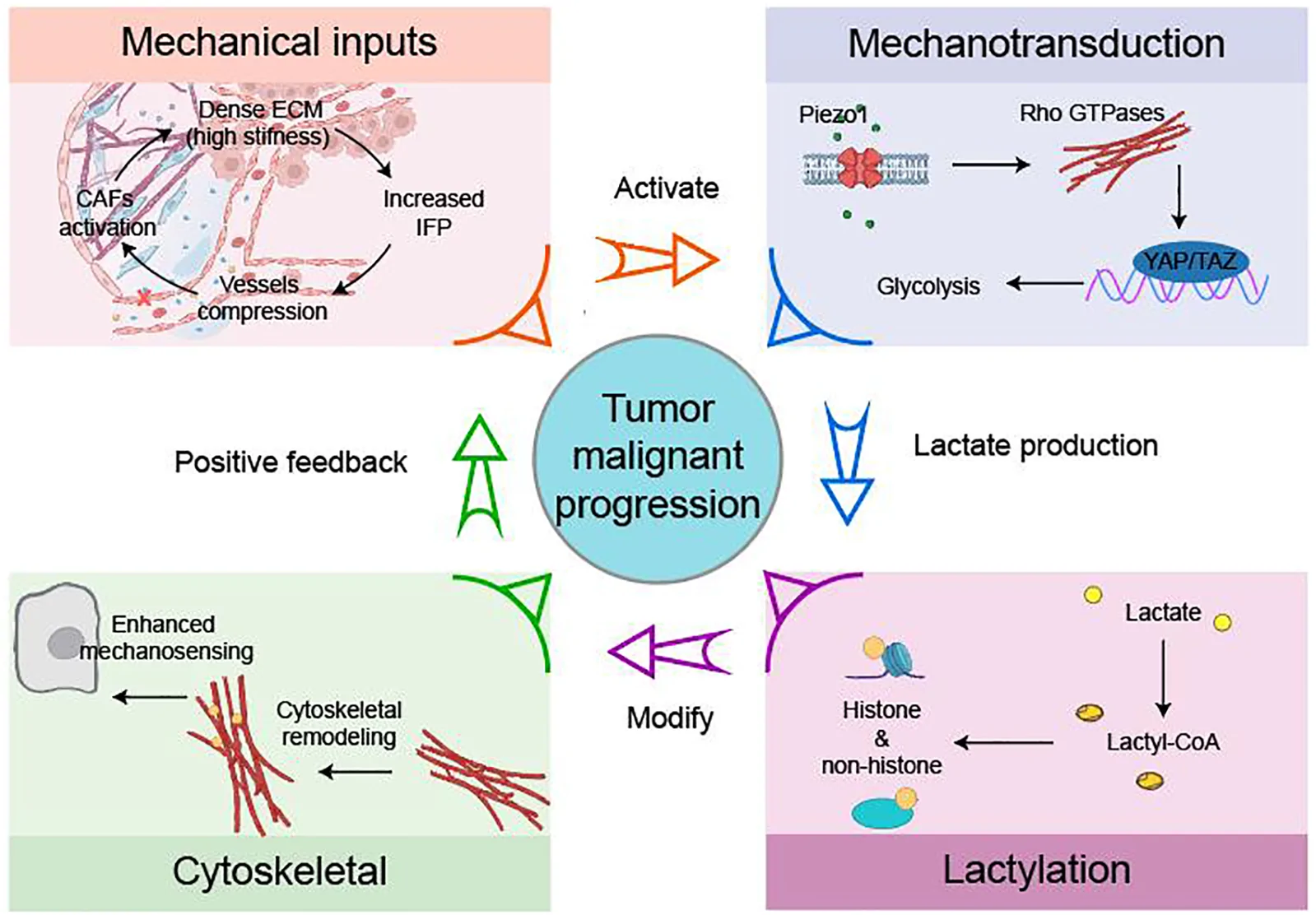
The mechano-metabolic-lactyl positive feedback loop driving tumor malignant progression. A self-reinforcing vicious cycle among mechanical cues, metabolic reprogramming, and post-translational modifications continuously fuels tumor evolution (1). Mechanical inputs: Aberrant physical signals in the TME, such as dense ECM, increased IFP, and CAFs activation, provide initial mechanical stress (2). Mechanotransduction: These inputs activate cell surface mechanosensors (e.g., Piezo1) and intracellular hubs (Rho GTPases), which subsequently drive YAP/TAZ nuclear translocation to induce metabolic reprogramming, particularly enhanced glycolysis (3). Lactylation: The Warburg effect results in massive lactate production, which is converted to lactyl-CoA to serve as a donor for the lactylation of both histone and non-histone proteins (4). Cytoskeletal remodeling: Non-histone lactylation directly modifies cytoskeletal components, leading to cytoskeletal remodeling and enhanced cellular mechanosensing. Ultimately, this heightened mechanosensitivity and contractility feed back into the TME to exacerbate mechanical abnormalities, thereby locking the tumor cells in an aggressive, pro-metastatic state.

## 5 Execution of metastatic cascades

### 5.1 Mechanically/lactylation-driven EMT program

EMT is a critical molecular program through which tumor cells acquire migratory and invasive capabilities and initiate the metastatic cascade. Aberrant mechanical signals in the TME—including increased matrix stiffness, shear stress, and hypoxia—regulate EMT not only through classical mechanotransduction pathways (e.g., YAP/TAZ, Rho-ROCK) but also by inducing lactate accumulation and lactylation, which directly remodel the EMT transcriptional network and cytoskeleton (257–260).

#### 5.1.1 Direct regulation of EMT by matrix stiffness and mechanical signals

Matrix stiffness, as an independent physical signal, can promote EMT through multiple routes. In cervical cancer, high matrix stiffness activates YAP via Pin1-mediated non-canonical Hippo signaling, promoting EMT and cell migration (261). Disruption of mechanical homeostasis is a trigger for EMT. During tumorigenesis, cancer cells alter their mechanical properties in a direction opposite to the environment, creating an imbalance of mechanical stress inside and outside the cell that drives EMT (259). In ovarian cancer, soft matrices paradoxically enhance the malignant phenotype of metastatic cells—including EMT morphological changes—through the Rho-ROCK pathway (262). Low shear stress combined with altered ECM composition can synergistically induce endothelial-to-mesenchymal transition (EndMT), upregulating Snail and α-SMA and promoting tumor-endothelial interactions (263). TNFα-induced inflammatory EMT is accompanied by cytoskeletal reorganization from a cortical to a stress-fiber pattern, with cell stiffness first decreasing and then partially recovering (264). These studies indicate that the type and intensity of mechanical signals exert bidirectional regulation on EMT.

#### 5.1.2 EMT transcription factors and cytoskeletal remodeling

Core EMT transcription factors (Snail, Twist1, SOX4, etc.) not only regulate gene expression but also directly alter cellular mechanical properties. In head and neck squamous carcinoma, high Snail expression increases nuclear longitudinal strain by enhancing cell traction forces and softening nuclear stiffness, thereby promoting cell invasion in confined environments (265). In breast cancer, TRPM7 regulates *SOX4* expression through tension, maintaining the mesenchymal phenotype. *SOX4* expression is inversely regulated by cytoskeletal tension and matrix stiffness (266). Twist1 upregulates cytoskeletal stiffness and traction forces through its conserved Twist box domain, promoting prostate cancer cell migration, invasion, and anoikis resistance, twhich domain is essential for EMT and metastasis (267). In head and neck cancer, EMT significantly reduces intracellular stiffness, and the reduction in stiffness is negatively correlated with invasive capacity in 3D environments, knockdown of *Twist1* or *Snail* increases intracellular stiffness (268). These findings indicate that EMT transcription factors directly endow cancer cells with the ability to penetrate physical barriers by regulating cytoskeletal and nuclear mechanical properties. Furthermore, cell shape and nuclear deformation themselves are regulators of EMT. Chimeric micropattern studies have shown that cell elongation and nuclear indentation upregulate EMT in a process dependent on actin contractility and mechanotransduction (269). PRELP binds to integrin α5 to activate the FAK/AKT pathway, reducing cell stiffness and adhesion and promoting colorectal cancer EMT and metastasis (270). TAGLN, as a stiffness-responsive biomarker, promotes metastasis in colorectal cancer through ECM remodeling and EMT (271). The CAR receptor cooperates with integrins, and stiffness-regulated phosphorylation promotes lung cancer cell adhesion and invasion (272). LOXL2, independent of ECM stiffness, promotes breast cancer lung metastasis by upregulating Snail and cytokines (273). EGCG inhibits EMT by increasing cell stiffness and suppressing *vimentin*/*Slug* expression (274). The differential mechanical properties of intermediate filaments (keratin vs. vimentin) also affect cell migratory behavior (275). Computational models further reveal how ECM stiffness and geometric constraints regulate cell cluster scattering and EMT through cell-cell junctions (276). Multiple histone lactylation sites (e.g., H3K18la, H3K9la, H3K14la, H3K27la, etc.) have been shown to activate the expression of pro-metastatic genes, promoting EMT, cell migration, invasion, and distant colonization.

#### 5.1.3 Molecular mechanisms of lactylation-driven EMT

Lactylation exerts multi-level regulation in the EMT process. In gallbladder cancer, lactylation of the transcription factor YY1 at lysine 183 (K183) enhances its transcriptional activation of the downstream target gene *FBXO33*, promotes p53 degradation through polyubiquitination at K291/K292, thereby inhibiting p53 function and promoting EMT and tumor metastasis (15). Under hypoxia, lactylation of PRMT1 at lysines 134/145 (K134/K145) enhances its methyltransferase activity, catalyzing symmetric dimethylation of vimentin at arginine 64 (R64). This modification drives proper vimentin filament assembly and cytoskeletal remodeling, conferring a mesenchymal phenotype and enhanced migratory capacity on tumor cells, promoting lung metastasis in breast cancer (207). In addition, delactylation of lactate dehydrogenase B (LDHB) at K58 suppresses STAT1 signaling, upregulates *SLC7A11* expression, enhances glutathione synthesis and ferroptosis resistance, and promotes metastatic progression in gastric cancer (277). Although this mechanism primarily involves metabolic adaptation, ferroptosis resistance is closely linked to the EMT phenotype.

In summary, mechanical forces (matrix stiffness, shear stress, hypoxia) and lactylation are deeply coupled in the EMT program. Mechanical abnormalities drive metabolic reprogramming and lactate accumulation, and lactylation directly modifies transcription factors (YY1), epigenetic regulators (PRMT1), and metabolic enzymes (LDHB), ultimately co-activating the Snail, Twist1, SOX4 EMT transcriptional networks and cytoskeletal reprogramming, providing the molecular execution basis for tumor cell detachment from the primary site, invasion into surrounding tissues, and distant dissemination (The integrated molecular mechanisms by which lactylation drives EMT and metastatic progression are synthesized in **Figure 6**). Targeting lactylation (e.g., inhibiting PRMT1 or YY1 lactylation) or mechanical signaling pathways (e.g., Pin1/YAP) may represent new strategies for blocking EMT and tumor metastasis.

**Figure 6:**
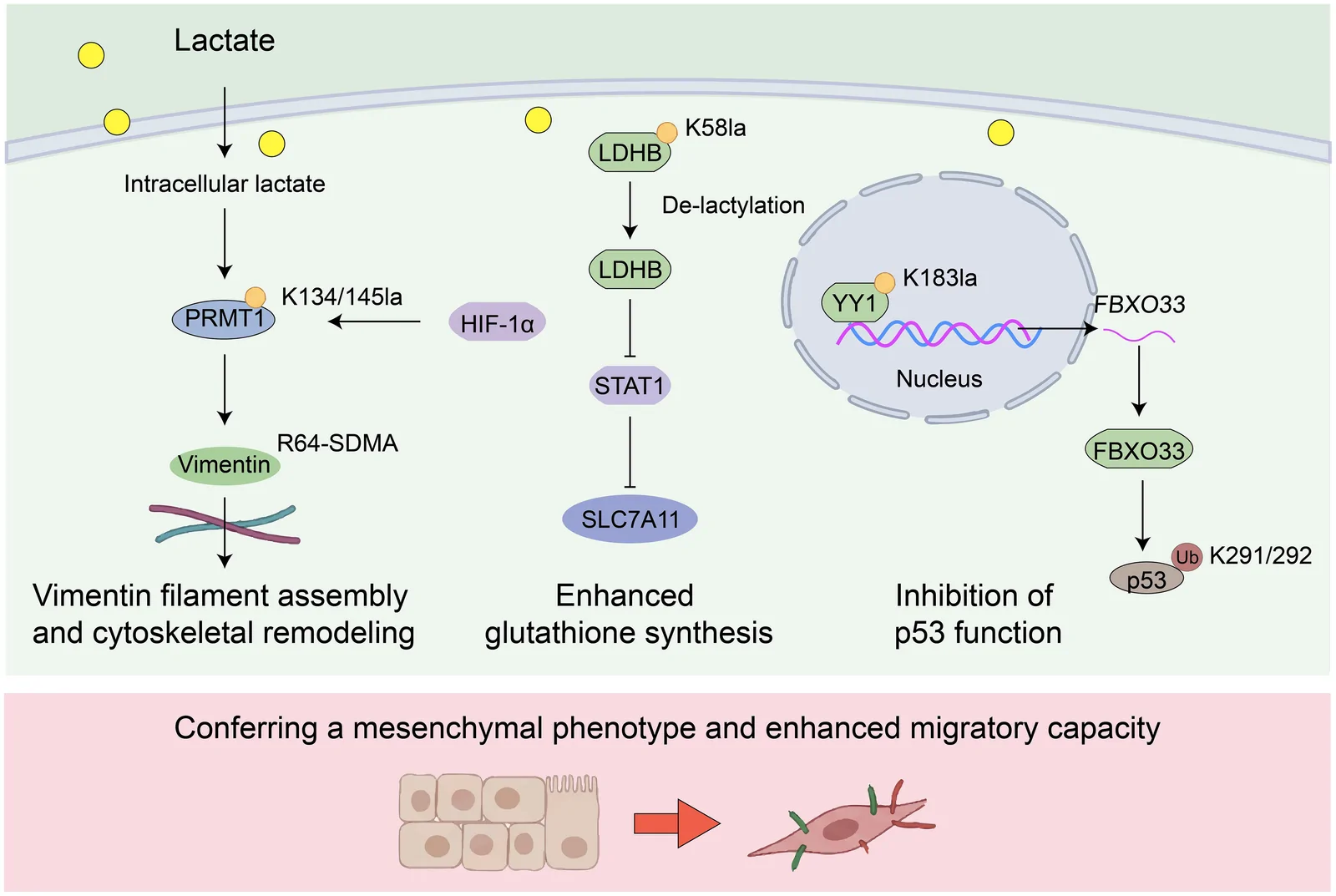
Molecular mechanisms of lactylation-driven EMT and metastatic progression. Elevated intracellular lactate levels trigger diverse non-histone lactylation events that orchestrate the EMT process through three major axes (1). Protein arginine methyltransferase 1/Vimentin(PRMT1/Vimentin) axis: Hypoxia-induced lactylation of PRMT1 enhances its methyltransferase activity, catalyzing the symmetric dimethylation of Vimentin to drive filament assembly and cytoskeletal remodeling (2). LDHB/STAT1/SLC7A11 axis: Delactylation of LDHB suppresses STAT1 signaling, which downregulates SLC7A11 to enhance glutathione synthesis and confer ferroptosis resistance (3). YY1/*FBXO33*/p53 axis: Lactylation of the transcription factor YY1 promotes the transcriptional activation of *FBXO33*, which subsequently mediates the polyubiquitination (Ub, ubiquitination modification at lysine-291/292 residues) and degradation of p53, thereby inhibiting p53-dependent tumor suppression. Collectively, these lactylation-driven signaling pathways facilitate the acquisition of a mesenchymal phenotype and enhance the migratory and invasive capacity of tumor cells.

### 5.2 Invadopodia assembly and ECM degradation execution

The ability of tumor cells to breach the basement membrane and traverse the ECM barrier is key to their invasiveness, and this process depends on a specialized actin-rich structure—invadopodia. Invadopodia are actin-rich membrane protrusions formed by transformed cells in 2D or 3D environments, responsible for ECM remodeling and degradation, and are critical for tumor cell invasion and metastasis both *in vitro* and *in vivo* (278). Integrins, as transmembrane adhesion receptors, play multiple roles in invadopodia formation and function. They not only mediate cell-ECM adhesion but also participate in signal transduction and mechanotransduction. Studies have shown that integrin expression is altered in various tumors and directly affects invadopodia assembly efficiency and their interactions with the ECM (278).

The functional execution of invadopodia depends on their secretion and recruitment of various proteolytic enzymes, especially matrix metalloproteinases (MMPs). Among them, MMP-2 and MMP-9 are core effectors that degrade basement membrane components (e.g., type IV collagen). Although studies in stem cell differentiation have shown that the expression and activity of MMPs such as MMP-9 are regulated by matrix stiffness and integrin signaling (279), direct evidence for how lactylation directly regulates MMPs or invadopodia assembly is still lacking. Given that lactylation has been shown in previous sections to regulate cytoskeletal proteins (e.g., vimentin, CFL1) and transcription factors (e.g., YY1, Snail), it is plausible that lactylation may indirectly participate in invadopodia maturation and function by influencing cytoskeletal dynamics and the EMT program. Future studies should further clarify whether mechanical signals and lactylation synergistically regulate MMP transcriptional activation, zymogen activation, and invadopodia formation efficiency, thereby fully resolving the execution mechanism of ECM degradation by tumor cells.

## 6 Discussion

This review provides an integrative framework for understanding how physical abnormalities in the TME drive metastasis. However, the molecular details of this loop—especially how different mechanical signals differentially activate specific lactylation events, the spatiotemporal division of labor between histone and non-histone lactylation, and the substrate selectivity of the lactylation enzyme system—remain to be elucidated. From a therapeutic perspective, targeting Piezo1, YAP/TAZ, LDHA, or lactylation “writers/erasers” holds promise, but challenges such as functional redundancy of target proteins and tumor-specific delivery must be addressed. Future efforts should focus on dissecting the spatiotemporal dynamics of the loop, developing bifunctional inhibitors that simultaneously intervene in mechanosensing and lactylation, and exploring the feasibility of lactylation as a biomarker for patient stratification, so as to translate these fundamental discoveries into anti-metastatic clinical strategies.
